# Fast-Onset Diffuse Interstitial Lung Disease in Anti-MDA5 Antibodies-Associated Amyopathic Dermatomyositis

**DOI:** 10.3390/clinpract11020035

**Published:** 2021-04-22

**Authors:** Houari Aissaoui, Kinan Drak Alsibai, Naji Khayath

**Affiliations:** 1 Department of Medicine, Pneumology Unit, Cayenne Hospital Center Andrée Rosemon, F-97306 Cayenne, France; 2 Department of Pathology and Centre of Biological Resources (CRB Amazonie), Cayenne Hospital Center Andrée Rosemon, F-97306 Cayenne, France; Kinan.drak.alsibai@gmail.com; 3 Centre of Biological Resources (CRB Amazonie), Cayenne Hospital Center AndréeRosemon, F-97306 Cayenne, France; 4 Chest Diseases Department, Strasbourg University Hospital, F-67000 Strasbourg, France; naji.khayath@chru-strasbourg.fr

**Keywords:** anti-MDA5 antibodies, dermatomyositis, rapidly progressive interstitial pneumopathy, polyarthritis

## Abstract

Anti-MDA5 antibodies-associated amyopathic dermatomyositisis a rare autoimmune disease that involve polyarthritis, cutaneous and pulmonary manifestations. The development of rapidly progressing interstitial lung disease is a life-threatening complication. We report the case of a 45-year-old woman without medical history, who was addressed to the Pulmonary Department for a polyarthritis with dry cough and hypoxemic dyspnea. Initially there was neither cutaneous manifestation nor interstitial lung disease on chest CT scan. After a few days, the patient developed fatal acute respiratory failure with diffuse ground glass opacities. Identification of anti-MDA5 antibodies allowed establishing diagnosis, despite the fact that the first immunological assessment was negative. Corticosteroid bolus of 1 g for three days and immunosuppressive treatment by cyclophosphamide was only initiated at the acute respiratory distress syndrome stage. Given the rapidly unfavorable prognosis of this entity of amyopathic dermatomyositis, the testing for anti-MDA5 antibodies should be recommended in case of progressive pulmonary symptoms associated with joint signs in order to identify this disease at an early stage and to begin rapid and adequate management.

## 1. Introduction

Dermatomyositis is a type of idiopathic inflammatory myopathy characterized by skin and muscular lesions. This rare autoimmune disease is heterogeneous and may be associated with malignant pathology in its paraneoplastic form. In the amyopathic dermatomyositis (ADM) form, patients present few or no muscular manifestations, but lung involvement is common [1,2]. This pulmonary damage affects the vital prognosis of patients and is the main cause of morbidity [1]. In addition, the clinical manifestation and severity of lung involvement vary greatly from one individual to another.

A new phenotype of ADM has been recently identified as being linked to the identification of the anti-melanoma differentiation-associated protein 5 antibodies (anti-MDA5 Ab). This antibody is present in 5–30% of dermatomyositis patients [3].

Clinically, anti-MDA5 Ab-associated ADM is mainly associated with articular, cutaneous and pulmonary damage; muscular damage is minimal or absent [4]. Skin lesions include keratotic papules on the fingers and palms, Gottron papules on the backs of the hands and extension surfaces of the knees and elbows, “mechanic’s hands”, peri-ungual skin ulcers, endobuccal ulcers and/or alopecia [5,6,7].

At the articular level, the main lesion is a symmetrical arthritis that tends to affect the small joints and may mimic rheumatoid arthritis [5,6]. These patients are known to develop rapidly progressive diffuse interstitial lung disease with a poor prognosis [8]. The chest CT scan usually shows nonspecific interstitial pneumonia lesions [4].

Several studies have shown that there is a relationship between the high level of theanti-MDA5Ab and the severity of the disease [9], the resistance to immunosuppressive treatments [10] and the risk of developing rapidly progressive lung disease [11,12] with a high mortality rate [13,14].

## 2. Case Report

This study reports the case of a 45-year-old patient, originally from the Maghreb, who has never smoked, a stay-at-home mother with no particular exposure and no notable clinical history apart from grade 2 obesity. She was referred by the local pneumologist to the Department of Pneumology of our hospital for an assessment of hypoxemic dyspnea associated with polyarthritis. The patient developed a month earlier a dyspnea with a fleeting rash on the forearms and cleavage that disappeared within a few days. A week later, she reported the appearance of symmetrical arthralgia affecting the distal and proximal joints of all four limbs that persisted despite treatment with non-steroidal anti-inflammatory drugs. The overall condition was not altered. The respiratory signs (dry cough and dyspnea) appeared later with progressive aggravation; up to 4 of modified medical research council dyspnea scale (mMRC scale). No medication was administered prior to the onset of joint symptoms.

The clinical examination revealed crackling rales in the two pulmonary fields and nonspecific cutaneous and articular clinical signs like curled fingers without sclerodactyly or mechanics’ hands or digital hippocratism. The rest of the clinical examination was normal. Further tests showed hypoxemia with pressure of arterial oxygen (PaO2) in ambient air at 57 mmHg, pressure of arterial carbon dioxide (PaCO2) at 34 mmHg, and 82% ambient air saturation. The patient was treated with oxygen 2 L/min.

**Figure 1:**
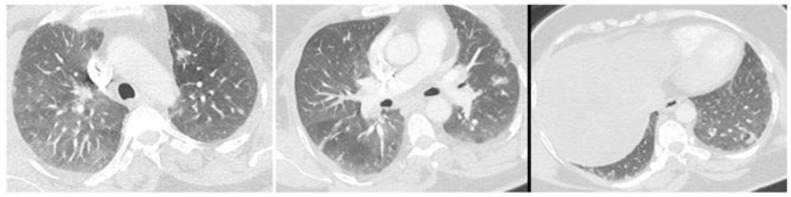
The chest CT scan performed three weeks after the onset of the respiratory sign joint signs, shows some pulmonary condensations.

The chest CT scan (Figure 1), which was performed three weeks after the onset of symptoms, revealed discrete pulmonary condensations.

A joint ultrasound showed bilateral knee arthritis, bilateral carpitis, discrete synovitis of the metacarpophalangeal articulations and extensor tenosynovitis of fingers bilaterally. A joint puncture of the right knee finds an inflammatory liquid, sterile and without crystals. Biologically, the rheumatoid factor, the Cyclic citrullinated peptide antibodies (anti-CCP) and the anti-nuclear antibodies and anti-neutrophil cytoplasmic antibodies (ANCA) were negative, as was the rest of the immunological analysis. The angiotensin converting enzyme (ACE) and the serum protein electrophoresis were normal. A discrete inflammatory syndrome with CRP at 16 mg/L was found. There was no sign of renal or liver damage and no proteinuria. The fibroscopy did not find any endo-bronchial lesions. The cytological formula of broncho-alveolar lavage (BAL) was without particularity (macrophages: 73%, lymphocytes: 12%, neutrophils: 13% and eosinophils: 2%). The bacteriological, virological and parasitic examinations of BAL were negative.

The patient’s condition was marked by a respiratory aggravation 5 days after admission with the appearance of a 37/min polypnoea at rest and a rapid increase in oxygen requirements (93% saturation under 15 L/min of oxygen in a high concentration mask) associated with persistent fever and a slight worsening of the inflammatory syndrome, whereas procalcitonin was negative. Broad-spectrum antibiotic therapy with piperacillin-tazobactam and ciprofloxacin was administered in the hypothesis of a nosocomial infection.

**Figure 2:**
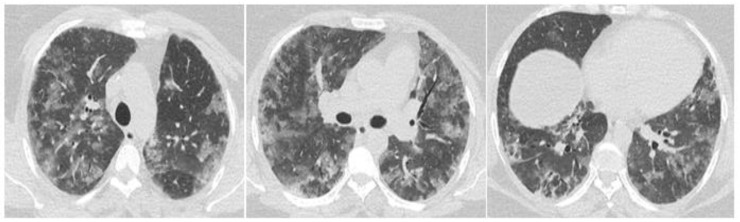
The chest CT scan performed at the time of sudden respiratory degradation, shows a rapidly progressive interstitial lung disease with the appearance of diffuse and extensive ground glass opacities.

A new chest CT scan was performed, finding extensive diffuse and bilateral ground glass opacities without reticulation, which did not spare the subpleural region (Figure 2). Corticotherapy by prednisolone at 1.5 mg/kg was immediately administered in addition to the antibiotic treatment. The trans-thoracic ultrasound ruled out heart failure.

The immunological analysis was enhanced by a large panel of extractable nuclear antigen (anti-ENA) antibodies. The results obtained four days later revealed the presence of anti-MDA5 Ab (+); therefore, the diagnosis of ADM was retained.

A new skin examination revealed a discreet peri-nail erythema and micro-papules on the posterior surface of the hands that were not initially present.

The patient’s respiratory degradation was severe; in less than 36 h, high-flow oxygen therapy became insufficient with a PaO2 at 59 mmHg under Optiflow with fraction of inspired oxygen (FiO2) at 1. Faced with this acute respiratory distress syndrome (ARDS), an orotracheal intubation was performed and the patient was transferred to the intensive care unit for mechanical ventilation.

The ARDS table attributed to an exacerbation of the anti-MDA5Ab (+) ADM immediately resulted in the immediate initiation of a more aggressive anti-inflammatory treatment with three boluses of 1 g prednisolone relayed by intravenous cyclophosphamide (750 mg/m^2^), combined with 1 mg/kg/day prednisolone. A new BAL was performed to eliminate pneumocystis.

On the respiratory level, the evolution was marked by infectious and iatrogenic complications (pneumopathy acquired under mechanical ventilation), with precarious stabilization at the rate of 100% FiO2 mechanical ventilation, alternation of ventral and dorsal decubitus and treatment with NO. Subsequently, respiratory function was again deteriorating, leading to the death of the patient by hypoxic cardiorespiratory arrest after three weeks of admission to intensive care.

## 3. Discussion

The case reported in this study highlights the discrepancy between the seriousness of the anti-MDA5 Ab-associated ADM disease and the initial clinical presentation, which was not specific. The absence of muscular damage, with joint lesions expressed by diffuse arthralgia but minimal arthritis, underlining the importance of early joint ultrasound to confirm the diagnosis, in particular by searching for tenosynovitis, which is an early lesion [15]. It is also important to carry out a very meticulous examination of the hands in search of signs of orientation of ADM. In our case report, the lesions were really discrete, not initially observed, or of late onset. However, the onset of dry cough followed the rash of the forearms and cleavage and the onset of arthralgia were alarming. Hypoxemia was moderate in relation to the patient’s grade 2 obesity. It was therefore mainly the rapid worsening of the dyspnea, which progressed over three weeks, that led to a chest CT scan and then hospitalization ten days after the CT scan was performed.

The non-specific characteristics of this dermatomyositis have led to a delay in diagnosis and a rapid evolution of lung damage. Several studies have highlighted the value of early treatment to improve the prognosis of ADM [10,16]. In our patient, the initial hypothesis was that of respiratory impairment of a possible rheumatoid arthritis. However, in the search for specific myositis antibodies, anti-nuclear antibodies, rheumatoid factor and anti-CCP were negative. While awaiting the biological and immunological results, the treatment was probabilistically based on expanded antibiotic therapy and corticosteroid therapy. Bolus doses of 1 g prednisolone and intravenous cyclophosphamide therapy were administered only at the ARDS stage, the choice having been made by analogy with the treatment of idiopathic pulmonary fibrosis exacerbation. Ikeda et al. have reported a series of refractory interstitial lung diseases developed in anti-MDA5 Ab-associated ADM with very high mortality rate despite intensive treatment [17].

The treatment of ADM is not yet standardized, it is based on corticosteroid therapy at a high dose of 1 mg/kg of prednisone, combined with immunosuppressants or used as a second line [18]. Some centers used at the onset of the disease the combination of triple therapy: corticosteroids, cyclophosphamide, and a calcineurin inhibitor (cyclosporin A or tacrolimus) [17,19]. Interestingly, a recent study has suggested that rituximab may be a useful therapy for anti-MDA5 Ab (+) ADMs associated with rapidly progressing interstitial lung disease. However, lung infection in this case is the major risk [20].

Gono et al. showed that there is a relationship between serum ferritin levels and the severity of interstitial lung injury in dermatomyositis, and that survival is lower in the group with ferritin levels greater than or equal to 1500 ng/mL [21]. However, in our case, the serum ferritin level was not very high (559 ng/mL).

Other prognostic markers such as elevated level of aspartate transaminase (AST) or gamma-glutamyl transferase (γ-GT), elevated CD4+/CD8+ ratio in BAL and the predominance of under-pleural ground glass opacities have been proposed [17]. In our case, the AST was less than two times the normal value at admission and did not increase to four times until ARDS. The rest of the liver exam was strictly normal and the pleural ground glass opacities appeared only at the late stage of disease.

## 4. Conclusions

Pulmonary involvement in anti-MDA5 Ab-associated ADM affects the vital prognosis of patients in the short and medium term, especially with the occurrence of rapidly progressive diffuse interstitial lung disease that responds poorly to conventional therapies. For this reason, it is necessary to quickly consider the diagnosis of ADM when there is an association between progressive respiratory signs, cutaneous and polyarticular involvement to identify this disease at an early stage and to begin rapid and adequate management. In addition, anti-MDA5 Ab testing should be considered in case of doubt of such a diagnosis due to the evolutionary and gravity characteristics of this entity.

## Data Availability

Not applicable.
